# Development and Comparative In Vitro and In Vivo Study of BNN27 Mucoadhesive Liposomes and Nanoemulsions for Nose-to-Brain Delivery

**DOI:** 10.3390/pharmaceutics15020419

**Published:** 2023-01-27

**Authors:** Maria Kannavou, Kanelina Karali, Theodora Katsila, Eleni Siapi, Antonia Marazioti, Pavlos Klepetsanis, Theodora Calogeropoulou, Ioannis Charalampopoulos, Sophia G. Antimisiaris

**Affiliations:** 1 Laboratory of Pharmaceutical Technology, Department of Pharmacy, University of Patras, 26510 Rio, Greece; 2 Foundation for Research and Technology Hellas, Institute of Chemical Engineering Sciences, FORTH/ICE-HT, 26504 Rio, Greece; 3 Department of Pharmacology, Medical School, University of Crete, 71003 Heraklion, Greece; 4 Institute of Molecular Biology & Biotechnology (IMBB), Foundation for Research and Technology-Hellas (FORTH), 70013 Heraklion, Greece; 5 Institute of Chemical Biology, National Hellenic Research Foundation, 48 Vassileos Constantinou Avenue, 11635 Athens, Greece

**Keywords:** BNN27, intranasal delivery, mucoadhesive formulations, chitosan, LIPs, nanoemulsions

## Abstract

Intranasal administration offers an alternative and promising approach for direct nose-to-brain delivery. Herein, we developed two chitosan (CHT)-coated (and uncoated) nanoformulations of BNN27 (a synthetic C-17-spiro-dehydroepiandrosterone analogue), liposomes (LIPs), and nanoemulsions (NEs), and compared their properties and brain disposition (in vitro and in vivo). LIPs were formulated by thin film hydration and coated with CHT by dropwise addition. BNN27-loaded NEs (BNEs) were developed by spontaneous emulsification and optimized for stability and mucoadhesive properties. Mucoadhesive properties were evaluated by mucin adherence. Negatively charged CHT-coated LIPs (with 0.1% CHT/lipid) demonstrated the highest coating efficiency and mucoadhesion. BNEs containing 10% *w*/*w* Capmul-MCM and 0.3% *w*/*w* CHT demonstrated the optimal properties. Transport of LIP or NE-associated rhodamine-lipid across the blood–brain barrier (in vitro) was significantly higher for NEs compared to LIPs, and the CHT coating demonstrated a negative effect on transport. However, the CHT-coated BNEs demonstrated higher and faster in vivo brain disposition following intranasal administration compared to CHT-LIPs. For both BNEs and LIPs, CHT-coating resulted in the increased (in vivo) brain disposition of BNN27. Current results prove that CHT-coated NEs consisting of compatible nasal administration ingredients succeeded in to delivering more BNN27 to the brain (and faster) compared to the CHT-coated LIPs.

## 1. Introduction

Dehydroepiandrosterone (DHEA) is one of the most abundant neuroactive steroids. It is synthesized in the human adrenal cortex as well as in the brain by neurons and glia [1]. It is a multifaceted agent, interacting with steroid and neurotransmitter receptors, and acts as an endogenous precursor for the biosynthesis of androgens, estrogens, and their metabolites [2]. It is known that DHEA increases the effects of the excitatory neurotransmitter glutamate, decreases the inhibitory neurotransmitter γ-aminobutyric acid (GABA), and stimulates the release of acetylcholine (Ach) in the hippocampus. Interestingly, all of the above-mentioned neurotransmitters have altered levels in patients with depression and stress-related disorders, suggesting the potential use of neurosteroids as therapeutic tools [1]. However DHEA’s metabolism to estrogens and androgens results in serious side-effects that limit its applicability as a long-term therapeutic agent despite its proven neuroprotective activity.

A new family of small synthetic C17-spiro DHEA derivatives named microneurotrophins have been synthesized [3] to mimic endogenous neurotrophin effects through the activation of specific receptors. *BNN27* [3β,21-dihydroxy-17*R*,20-epoxy-5-pregnene], a BBB-permeable C17-spiroepoxy DHEA derivative, with agonistic activity against the neurotrophin receptors ΤrkA and p75NTR, exhibits anti-apoptotic properties in vitro and in vivo [3,4,5]. Its main advantage compared to DHEA is the lack of hormone-receptor activation while preserving its anti-apoptotic and neurotrophic properties [6], therefore, it can be considered as a novel lead compound for developing non-toxic, BBB-permeable, neurotrophin receptor agonists and antagonists for therapeutic applications in neurodegenerative diseases, brain trauma, and neuropathic pain [7]. In order to enhance the highly interesting profile of BNN27 as a CNS therapeutic, nose-to-brain delivery was considered herein as a brain targeting strategy [8,9,10].

In most cases, treatments for CNS disorders are administered parenterally, reducing drug effectiveness and potency. Even if the lipophilicity of the drug does not impede its accessibility to the brain through the circulation, systemic clearance significantly reduces the drug bioavailability [8]. In addition, plasma protein binding delays delivery to the brain through circulation, and the peripheral side effects of systemic administration routes have triggered the hunt for alternative routes that deliver drugs directly to the brain. The intranasal route has emerged as a powerful strategy to circumvent the BBB [9,10], by delivering drugs directly to the brain through the nasal cavity via olfactory neurons. This pathway is associated with enhanced safety, increased patient compliance, remarkable ease of administration, rapid onset of action as well as minimized systemic exposure [9]. The design of suitable nose-to-brain formulations with enhanced mucoadhesive properties, large surface contact area and penetration enhancement, is necessary for successful intranasal drug delivery [10].

Herein, we prepared two different formulation types, LIPs and nanoemulsions with the optimal physicochemical and mucoadhesive properties, and compared them for their potential to deliver BNN27 to the brain following nasal administration. To the best of our knowledge, this is the first time that such mucoadhesive formulations have been compared for their brain disposition after intranasal delivery.

## 2. Materials and Methods

1,2-Distearoyl-sn-glycerol-3-phosphatidylcholine (PC) and 1,2-distearoyl-sn-glycero-3-phospho-(19-rac-glycerol) (sodium salt) (PG), and Lissamine Rhodamine B phosphatidylethanolamine or Rhodamine-lipid (RHO), were purchased from Avanti Polar Lipids (Alabaster, AL). Capmul MCM was received as a gift sample from Abitec Corporation Limited (Columbus, OH, USA). Labrafac Lipophile WL 1349, Labrafac PG, and Transcutol HP were received as gift samples from Gattefosse (Lyon, France). Tween 80 was purchased from Fisher BioReagents and Tween 20 from BioChemica UK Ltd. Carbopol 974P was kindly provided by Chemix SA (Athens, Greece). Cholesterol, mucin from porcine stomach Type III bound sialic acid 0.5–1.5% (partially purified powder), low molecular weight chitosan (LMW-CHT, with a molecular weight of 50–190 kDa and 75–85% deacetylated) and medium molecular weight chitosan (MMW-CHT, with a molecular weight between 190–310 kDa and 75–85% deacetylated), and all other excipients for the nanoemulsion preformulation studies were purchased from Sigma-Aldrich or Merck. BNN27 was kindly provided by Bionature Ltd.

For the quantification of BNN27, an enzymatic method—a cholesterol kit purchased from Biotechnological applications LTD (Athens, Greece)—was used. All other chemicals were of analytical quality and were purchased from Sigma-Aldrich or Merck (Darmstadt, Germany).

### 2.1. Quantification of BNN27 Concentration in Formulations

For the measurement of BNN27 loading in the various formulations during formulation development, as a routine everyday quantification method, the CO/PAP enzymatic method [11,12], which is used for the measurement of blood cholesterol (Cholesterol Determination Kit, Biotechnological Applications), was applied (due to the structural similarity of BNN27 with cholesterol). For this, appropriate BNN27 calibration curves were constructed by preparing BNN27 standard solutions of known concentrations ranging between 0 and 150 ppm in ethanol. A sample of 100 µL from each solution was mixed with 100 µL of PBS (or empty LIPs, in order to see if the presence of LIP components disturbed the BNN27 measurement) and 1 mL of the Cholesterol Measurement Kit reagent was added. After mixing and 15 min incubation at 37 °C, the optical density at 510 nm was measured by a Shimatzu UV-1205 spectrophotometer.

The measurement of BNN27 in ΝΕs or solutions of NE ingredients is not possible with this method due to interactions between the NE ingredients and reagent. Thereby, the BNN27 extraction was applied for the measurement of BNN27 in NEs or in NE ingredients (oils, surfactants and co-surfactants). For this, 1 mL of BNN27-containing NEs or solutions was vigorously mixed (by vortex) with 2 mL chloroform for (at least) 2 min. Then, after complete separation of the two phases, the aqueous phase was removed and the organic was evaporated. The drug was re-suspended in 200 μL of ethanol and mixed with 1 mL of reagent. After incubation at 37 °C for 15 min, the sample OD-510 nm was measured.

The BNN27 content was calculated from a calibration curve that was conducted by the same method (applied in each case) using known amounts of BNN27 mixed with empty LIPs, blank NEs, or NE components.

### 2.2. Preparation and Characterization of LIP and Chitosan Coated LIPs

#### 2.2.1. Preparation of BNN27-Loaded LIPs

MLV LIPs composed of PC or PC/PG at 9:1 (mol/mol) and loaded with BNN27 were prepared by the thin-film hydration method [13,14]. For this, the lipid(s), together with the drug, was dissolved in the CHCl_3_/MeOH (2:1 *v*/*v*) mixture in a round bottom flask and a thin lipid film was formed by rotor-evaporation of the organic solvents. The thin lipid film was hydrated with PBS, pH 7.40. Different amounts of BNN27 were included in the lipid solution in order to identify the maximum loading conditions. After the formation of multilamellar vesicles, their size was reduced by probe sonication (Sonics & Materials) in order to produce SUV LIPs. For this, 3–5 min of sonication with a tapered microtip was applied at 35% intensity until a clear dispersion was produced. Following sonication, the SUV LIPs were incubated at room temperature for 1 h in order to anneal any structural defects.

Separation of LIPs from the non-encapsulated BNN27 was achieved by centrifugation (15,000 rpm for 30 min) and supernatant filtration through 0.45 μm pore filters, in order to separate the LIP dispersions from any non-incorporated and precipitated BNN27. LIPs were stored at 4 °C before use.

#### 2.2.2. Preparation of Chitosan-Coated LIPs

For the LIP coating, two types of CHT were used: low molecular weight (LMW) and medium molecular weight (MMW). CHT solutions of different concentrations were prepared in isotonic acetate buffer (pH = 4.40). Then, 0.5 mL of the LIP dispersion (in PBS) was mixed with the dropwise addition of an equal volume of the appropriate CHT solution to give CHT/lipid (*w*/*w*) ratios of 0.1, 0.3, or 0.5, under continuous stirring for 1 h at room temperature [13], followed by overnight incubation at 4 °C [15]. Then, CHT-coated LIPs were harvested from the reaction mixture by centrifugation at 15,000 rpm for 15 min. The supernatant (non-adhered CHT) was removed and the pellet, consisting of CHT-coated LIPs, was re-suspended in PBS.

For coating efficiency determination, the phospholipid content of the samples was measured by the Stewart assay and compared to the total phospholipid (before centrifugation), as previously reported [13,16]. The coating efficiency (%) was calculated from the equation:

Coating Efficiency (CE) % = LPRE (lipid-in-precipitate)/LTOT (Total Lipid) × 100(1)

#### 2.2.3. Physicochemical Properties of LIPs

The size distribution (mean hydrodynamic diameter and polydispersity index) and ζ-potential of the LIP dispersions were measured by dynamic light scattering (DLS) and laser Doppler electrophoresis (LDE), respectively, on a Nano-ZS (Nanoseries, Malvern Instruments), which measures the mass distribution of the particle size as well as the electrophoretic mobility of the dispersed particles. Measurements were made at 25 °C with a fixed angle of 173°. Sizes quoted are the *z*-average mean (d*z*) for the liposomal hydrodynamic diameter (nm). Calculation of ζ-potential (mV) was carried out by the instrument from electrophoretic mobility, which was measured in small volume disposable zeta cells and converted to zeta potential by in-built software that applies the Helmholtz–Smoluchowski equation. For measurements, samples were diluted to have a 0.4 mg/mL lipid concentration.

### 2.3. Preparation of BNN27-Loaded Nanoemulsion

In order to formulate BNN27-loaded nanoemulsions (BNEs), we followed the previously reported methodologies for the optimal formulations of nanoemulsions intended for nose-to-brain delivery of various drugs such as risperidone [17,18], rivastigmine [19], and quetiapine fumarate [20]. The selection of ingredients was based on the BNN27 solubility measurements, ternary phase diagrams (to select the optimal surfactant/co-surfactant ratio in the surfactant-co-surfactant mixture (Smix)), and the physicochemical properties/stability of the formulated NEs.

#### 2.3.1. Solubility of BNN27 in Potential NE Ingredients

In order to select the optimal materials, BNN27 solubility studies were carried out. The solubility of BNN27 was measured in three different oils that are commonly used in NEs [18,19,20,21], Capmul MCM, Labrafac Lipophile WL 1349, and Labrafac PG. Additionally three surfactants, Tween 20, Tween 80, and Cremophor EL RH40 as well as three co-surfactants, PEG 400, Transcutol HP, and a Transcutol HP/propylene glycol (1:1) mixture, were studied. For solubility determination, BNN27 was added as a solid powder in the different liquids or mixtures until a noticeable amount of the powder was not solubilized, and stirred for 24 h on a mechanical rocking shaker (Kisher Biotech) at room temperature (25.0 ± 2.3 °C). Afterward, only the samples that still had visible undissolved solid powder were further processed by centrifugation (10,000× *g* for 10 min) and the supernatants were diluted. Finally, BNN27 solubilized in the liquids was measured by the method described in detail above (Section 2.1).

#### 2.3.2. Optimization of NE Formulation

After the selection of the best oil and co-surfactant (based of BNN27 solubility), and due to the fact that two of the tested surfactants, Tween 20 and Tween 80, demonstrated similar (very high) ability to solubilize BNN27, both surfactants were studied with the scope to finally select the one that conferred NEs with the maximum stability. Since it is known from the relevant literature that the best surfactant/co-surfactant ratio in the case when Tween 80 is used (as surfactant) and Transcutol HP/propylene glycol (1:1) (as co-surfactant) is 4:2 (*w*/*w*) [18], we initially constructed similar ternary diagrams to find out whether the same optimal ratio also applies when Tween 20 is used as a surfactant. Additionally, since it has been reported that in NEs containing Tween 80 together with Transcutol HP/propylene glycol mixtures, the NE globule size is the lowest when the percent of Smix is 44% (*w*/*w*), and does not further decrease when increasing the Smix content [17,20], a number of NEs containing Tween 20 as the surfactant instead of Tween 80 and different Smix amounts were formulated and evaluated for the effect of the Smix amount on the corresponding NE’s globule size and transparency.

Finally, the globule sizes of the NEs constructed using Capmul MCM as the oil phase at 8% or 10% (*w*/*w*), and Tween 20 or Tween 80 as the surfactant (always mixed with Transcutol HP/propylene glycol (1:1) (as the co-surfactant) with 44% (*w*/*w*) Smix content), were compared in order to identify which of the two surfactants, Tween 20 or Tween 80, produced NEs with the lowest globule size and highest stability.

#### 2.3.3. Preparation of BNN27-Loaded NEs

After the identification of the optimal NE composition, BNN27-loaded NEs were formulated by the spontaneous emulsification (titration) method. For this, a saturated solution of BNN27 in Capmul MCM was prepared by adding 40 mg/mL BNN27 in the oil phase and applying magnetic stirring. Then, in the BNN27 Capmul MCM phase, the Smix (containing Tween 80 as surfactant) was added until a clear mixture was produced. Finally, H_2_O was added dropwise and stirred to produce clear NEs of BNN27. If all of the BNN27 is loaded with a 10% *w*/*w* oil phase, the NEs should contain 4 mg of BNN27 per mL of NE.

Mucoadhesive, chitosan (CHT) or carbopol (CAR) coated BNN27-loaded NEs (BNEs) were also prepared. For this, concentrated BNEs (using the minimum volume of the external phase) were initially prepared, and then mixed with the required volume of CHT or CAR aqueous solution to attain a final CHT or CAR concentration of 0.3% *w*/*w*. After the addition of CHT or CAR, the BNEs were allowed to homogenize by continuous stirring for 1 h.

#### 2.3.4. Physicochemical Properties and Stability of NEs

The quality and stability of the various NE or BNE formulations constructed were evaluated by dilution tests, centrifugation tests, measurements of pH, transmittance, globule size distribution, and ζ-potential. The dilution test was performed by diluting 1 mL of NEs to 100 mL with d.d. H_2_O, and applying optical observation of the NEs for clarity/turbidity. For the centrifugation test, NEs were centrifuged at 300× *g* for 15 min and examined by visual observation if they remained monophasic or if phase separation occurred. The pH of the NEs was measured in 5 mL samples placed in a 10 mL beaker by a pH meter (Consort P902). The transmittance percent (%T) of NEs at 650 nm was measured using a UV–VIS spectrophotometer. Finally, the globule size distribution and ζ-potential of the NEs were measured with the same methods described for LIPs (§ 2.2). For measurement, the NE samples were diluted 20 times in d.d. H_2_O. All measurements were performed in triplicate.

The stability of the NEs (coated and non-coated) was evaluated by applying all of the methods above-mentioned at various time points (1, 7, 14, 21, 30, 60 d) during storage (for up to 2M) at room temperature as well as at 4 °C.

### 2.4. Mucoadhesive Properties

The adsorption of mucin on the surface of LIPs or NEs was used as a method to assess the mucoadhesive properties of the BNN27-loaded LIPs and NEs [13].

For LIPs, 1 mL of mucin aqueous solution (0.5 mg/mL) was mixed (vortexed) with an equal volume of each LIP dispersion (lipid concentration at 2 mg/mL) at room temperature and the dispersions were centrifuged at 15,000 rpm for 30 min. Free mucin was measured in the supernatant. PC and PC/PG (negatively charged) LIPs were studied, before and after coating. The same protocol was used for the measurement of the mucoadhesive properties of NEs, after the NEs were diluted in order to eliminate any turbidity that would affect the measurements.

For measurement of the free mucin in the supernatants, the Bradford colorimetric method was used [22]. Samples (and standard solutions) were incubated for 20 min at 37 °C after the addition of the Bradford reagent, and then absorbance at 595 nm was measured (Shimatzu UV-1205 spectrophotometer). A mucin calibration curve was prepared by measuring the mucin standard solutions, and the mucin content of each sample was calculated from the calibration curve. Finally, the amount of mucin adsorbed on the samples was calculated as the difference between the total and free mucin.

### 2.5. Cell Culture Studies

#### 2.5.1. Cytotoxicity Assessment

Immortalized human brain microvascular endothelial cells (hCMEC/D3) as well as human embryonic kidney cells (HEK) were used. HEK cells were grown in a high glucose DMEM medium supplemented with 10% FBS and 1% antibiotic-antimycotic solution (Invitrogen, Carlsbad, CA, USA). The cells were cultured at 37 °C, 5% CO_2_/saturated humidity. The medium was changed every 2–3 days.

hCMEC/D3 cells (passage 25–35) were obtained under license from the Institut National de la Sante et de la RechercheMedicale, INSERM, Paris, France and grown in EndoGRO medium (Merck, Darmstadt, DE) supplemented with 10 mM HEPES, 1 ng/mL basic FGF (bFGF), 1.4 μM hydrocortisone, 5 μg/mL ascorbic acid, penicillin-streptomycin, chemically defined lipid concentrate, and 5% ultralow IgG FBS. All cultureware was coated with 0.1 mg/mL rat tail collagen type I (BD Biosciences, Franklin Lakes, NJ, USA).

The cytotoxicity of the liposomal and nanoemulsion samples toward the hCMEC/D3 and HEK cells was evaluated with the MTT assay. Briefly, 25,000 cells were seeded in collagen pre-coated 24-well plates and after overnight incubation, the medium was replaced with the amount of each sample required to confer 1 μM BNN27, and incubated at 37 °C and in 5% CO_2_ for 48 h. After completion of the cell/vesicle incubations, MTT solution was added in all samples and after 2 h (for HEK cells) or 4 h (for hCMEC/D3 cells), acidified isopropanol was used to dissolve the formazan crystals that were formed. Viable cells (%) were calculated based on the equation: (A620 sample-A620 background)/(A620 control-A620 background) × 100, where the A620 control is the OD-620 nm of untreated cells, and the A620 background is the OD-620 nm of MTT without cells.

#### 2.5.2. Cell-Monolayer Permeation Studies

For the monolayer studies, hCMEC/D3 cells were seeded on Transwell filters (polycarbonate six-well, pore size 0.4 μm; Millipore Merck, Darmstadt, DE) pre-coated with type I collagen, at 5 × 10^4^ cells/cm^2^. The detailed procedure followed, and the tests that were carried out to verify that the monolayer produced was intact (transendothelial electrical resistance measurements (TEER) and Lucifer yellow (LY) permeability calculation) are described in detail elsewhere [14,23].

The permeability of the samples was determined after the preparation of RHO-labeled nanoformulations. After adding the samples to the top of the permeation filter (0.2 μM RHO per filter), the transport of RHO was calculated by the fluorescence intensity measurements (540/585) of the samples taken from the basolateral portion at selected time points (10, 30, 60, 90, and 120 min). For the extraction of RHO-lipid from the formulations, the Folch method was applied, as previously reported [24].

### 2.6. Transmission Electron Microscopy (TEM)

LIPs (0.5–1 mg/mL) were re-suspended in 10 mM HEPES (to eliminate potential artifacts from phosphate salts) while NEs were diluted in water. Then, all types of samples were negatively stained with 1% phosphotungstic acid in dH_2_O (freshly prepared), washed three times with dH_2_O, drained with the tip of a tissue paper, and observed at 100,000 eV with a JEM-2100 (Jeol, Tokyo, Japan) transmission electron microscope (TEM) [25].

### 2.7. In Vivo Studies

For the in vivo study, C57BL/6J 8-week old mice were utilized. Animals were housed and maintained in a 12-h light/dark cycle and fed ad libitum. All procedures were performed according to the European Union policy (Directive 86/609/EEC) (carried out in compliance with Greek Government guidelines) and institutionally approved protocols (Veterinary Directorate of Prefecture of Heraklion (Crete) and FORTH ethics committee (License number: EL91-BIOexp-02)). Mice were anesthetized using an intraperitoneal injection of a ketamine (Nerketan 10, 100 mg/mL) and xylazine (Xylapan, 20 mg/mL) cocktail. Once the hind-limp was lost, the mice were fixed in the supine position, 25 µL of each formulation was administered to each mouse in 60 s intervals, via 1–2 µL dose alternatively into each nostril [26]. Animals were sacrificed 60 and 120 min after the completion of the administration. Brains were quickly dissected out of the cranium; excess blood was wiped off, and the brain was snap frozen until further processing.

After weighing, brain samples were homogenized in 300 µL ice-cold distilled water/methanol solution (25/75 *v*/*v*) and sonicated for 20 min at 4 °C. Next, three volumes of ice-cold acetonitrile were added, followed by sonication for 10 min and centrifugation at 14,000× *g*/15 min/4 °C. After an extra addition of 100 µL ice-cold acetonitrile to the supernatant, a 10 min-sonication and centrifugation at 14,000× *g*/15 min/4 °C were carried out. The supernatant was vacuum-dried at a SpeedVac without heating. Prior to analysis, samples were stored at −80 °C.

To quantify the BNN27 levels, the detection limit of the enzymatic method used for BNN27 quantification in the formulations (>2 ppm) was not low enough; therefore, a liquid chromatography mass spectrometry (LC-MSn) method was developed using deuteriated pregnenolone (pregnenolone 17,21,21,21-D4) as the internal standard (70 ng/mL). The analysis was performed on an LTQ-Orbitrap Velos mass spectrometer (MS) (Thermo Fisher Scientific, Bremen, Germany) connected to an Accela ultra-high-performance LC (UHPLC) system. An Acquity UPLC BEH C18 VanGuard pre-column (130 Å, 1.7 µm, 2.1 mm × 100 mm) coupled to an Acquity UPLC BEH C18 column (130 Å, 1.7 µm, 2.1 mm × 5 mm) was used. Quality control samples were prepared at three concentrations (low, medium, high) to monitor the instruments’ performance and chromatographic integrity over time. Monitoring occurred in positive ion mode. The standard curve concentration range was 1–2000 ng/mL (Y = 0.000546519 + 0.000897604∙X; R2 = 0.9673; W:1/x). The injection volume was set at 5 µL, and the mobile phase flow rate was set at 0.2 mL/min. Mobile phase solvents were A (95% H_2_O, 5% methanol, 0.1% formic acid) and B (methanol, 0.1% formic acid). The eluting gradient program was the following: 0–0.1 min (40% A, 60% B), 0.1–0.5 min (20% A, 80% B), 0.6–6.5 min (5% A, 95% B), 6.51–8.0 min (40% A, 60% B). Data were processed with Xcalibur software (version 2.1, Thermo Scientific, Waltham, MA, USA) and data analysis was conducted using the R programming language. In order to calculate the BNN27 levels in brain tissue, expressed as administered dose percent (ID%), the following formula was applied: ID%/g brain = (Xbrain/X in dose) × 100; where: Xbrain = BNN27 (mg) per g of weighted brain tissue, and X in dose = BNN27 (mg) in 25 µL solution for intranasal administration.

### 2.8. Statistical Analysis

All results were expressed as mean ± S.D from at least three independent experiments. Most data were analyzed by using one way ANOVA followed by the Bonferroni post hoc test. *p* < 0.05 was considered statistically significant for all comparisons. When more factors were compared, two-way ANOVA was performed. The significance of comparisons is presented in the graphs as: * *p* < 0.05, ** *p* < 0.01, *** *p* < 0.001, **** *p* < 0.0001.

## 3. Results and Discussion

### 3.1. BNN-Loaded LIPs and Chitosan-Coated LIPs

All calibration curves constructed both in the absence and presence of empty LIPs were linear; proving that the quantification method applied resulted in the accurate determination of BNN27 in LIPs (see Supplementary Materials Figure S1).

#### 3.1.1. BNN Loading in LIPs

**Figure 1:**
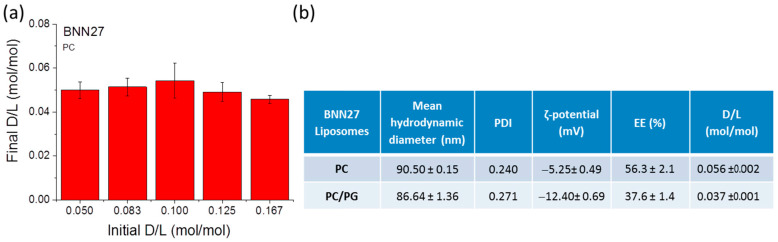
(**a**) Effect of increasing the initial amount of BNN27 (or D/L mol/mol ratio) on the amount of BNN27 loaded in PC small unilamellar LIPs. (**b**) Physicochemical properties of BNN27-loaded LIPs. Each value is the mean from at least three independent samples.

The BNN27 content of each LIP type was calculated from the appropriate calibration curve, after mixing equal volumes of the liposomal sample and pure ethanol, and applying the method described above. The results of studies carried out to optimize BNN27 loading in LIPs can be seen in Figure 1a. As seen, BNN27 loading could not confer a D/L (mol/mol) ratio higher than 0.056 that was realized when using an initial D/L ratio equal to 0.1 (mol/mol). Furthermore the loading efficiency was not significantly modified by using different initial D/L ratios, between 0.05 and 0.167.

Concerning the lipid membrane composition effect on BNN27 loading in LIPs, as seen from the results in Figure 1b, the addition of negative charged lipids (PG) resulted in a significant decrease in BNN27 incorporation into the LIPs. It is well-known that the composition of LIP lipid membranes determines the lipophilic drug partitioning/incorporation in LIPs, since these drugs are incorporated in the lipid membrane. Regardless of the decrease in BNN27 loading, the addition of the negative charge is very important for liposomal surface modification such as coatings with chitosan, as demonstrated previously and verified by the results presented in the following section.

From the Figure 1b results, the nanosize of both types (lipid membrane compositions) of BNN27 loaded LIPs was confirmed as well as their narrow size distribution. As anticipated, the addition of PG in the LIP membrane conferred a significant increase or the vesicle negative zeta potential.

#### 3.1.2. Coating of BNN27-Loaded LIPs with Chitosan

The coating efficiency of chitosan-LIPs (CHT-Lip), together with the physicochemical properties of the various LIP types prepared, is presented in Table 1. As demonstrated, the vesicle coating efficiency was substantially higher in the case of PC/PG LIPs compared to PC LIPs, irrespective of the amount/type of CHT used. This is in agreement with a previous report that the CHT coating of LIPs cannot be further increased above the 0.1 *w*/*w* chitosan/lipid ratio [13]. Indeed, when the medium molecular weight (MMW) chitosan was used, vesicles with the lowest size (1147 ± 86 nm) and polydispersity index (PDI = 0.447) were formed at 0.1 *w*/*w* CHT/lipid ratio. Higher CHT amounts resulted in the formation of liposomal clusters with large sizes and high PDI values. Furthermore, the vesicle ζ-potential value, which is used as a measure of the vesicles’ mucoadhesive capacity, was not significantly increased when higher than 0.1 CHT/lipid ratios were used (Table 1); thereby we continued our experiments with LIPs that were coated using 0.1 *w*/*w* CHT/lipid.

Uncharged PC vesicles had much lower CHT coating efficiencies (Table 1) compared to the negatively charged PC/PG LIPs. It was previously explained that in addition to the electrostatic interactions between positively charged chitosan and negatively charged LIPs, the coating process is regulated by a number of other mechanisms such as the formation of hydrogen bonds between the hydrogen of the polysaccharide and the nitrogen groups of the polar head of PC [27]. Nevertheless, differences between PC LIPs that were coated with LMW chitosan and MMW chitosan (concerning their size distribution and zeta-potential) were not significant, most probably due to the very low amount of CHT adhered on these non-charged vesicles. In contrast, the molecular weight of CHT had a more obvious effect when negatively charged LIPs were studied. Indeed, the variations in the mean hydrodynamic diameter of the LIPs and ζ-potential values, between vesicles that were coated with LMW CHT and MMW CHT, were statistically significant (*p* < 0.0001). The coating of vesicles with MMW chitosan resulted in increased liposomal size and ζ-potential values approx. by 1.5 times compared to the coating with LMW CHT, but most importantly, the vesicle zeta-potential values were similarly increased (Table 1). For the latter reason, MMW CHT coated LIPs were used in all of the following studies. Thereby, the LIPs used in the following studies were PC/PG/BNN27 CHT-LIPs with MMW CHT.

### 3.2. Development of BNN27-Loaded Nanoemulsions

#### 3.2.1. BNN27 Solubility Studies—Selection of NE Ingredients

**Figure 2:**
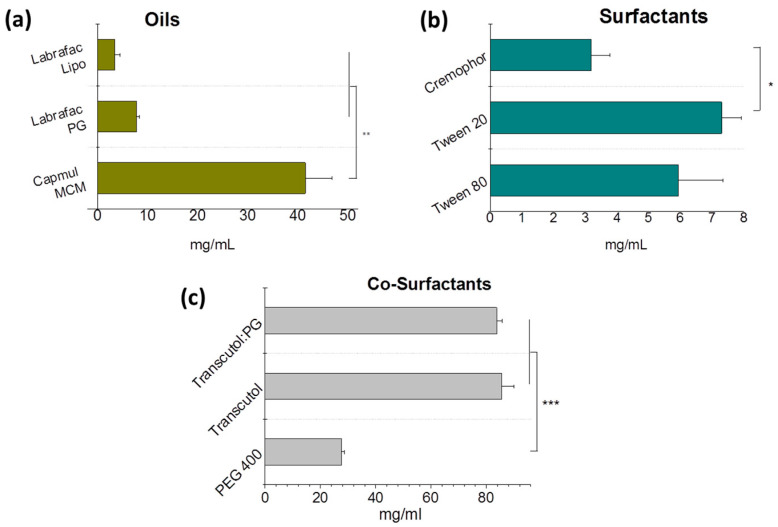
Solubility of BNN27 in various potential ingredients of NEs, expressed as mg/mL. Each value is the mean of at least three independent samples. (**a**) Solubility in oils. (**b**) Solubility in surfactants. (**c**) Solubility in co-surfactants. *: *p* ≤ 0.05; **: *p* ≤ 0.01; ***: *p* ≤ 0.001.

The solubility of practically insoluble (in water) BNN27 in selected potential ingredients of NEs is reported in Figure 2. As seen, between the oils tested (Figure 2a), the highest solubility was measured in Capmul MCM, so this oil was selected as the oil phase for NEs.

Concerning the solubility of BNN27 in surfactants (Figure 2b), the highest solubility was measured in Tween 20 and Tween 80; therefore, we selected to study both surfactants in order to select the surfactant that acquired the optimal NE properties.

Finally, between the potential co-surfactants tested (Figure 2c), very high BNN27 solubilities were found in both the Transcutol and Transcutol/propylene glycol (1:1) mixture. In fact, we selected to use the Transcutol/PG mixture, since it has been previously reported to impart high stability and lower globule size (compared to other co-surfactants) to the NEs [20].

#### 3.2.2. Selection of Optimal Surfactant and Composition for NE

The type of NE formed depends on the properties of the oil, surfactant, and co-surfactant. Many surfactants cannot lower the oil–water interfacial tension sufficiently to form NEs, so co-surfactant addition is necessary. Co-surfactants additionally ensure that the interfacial film is flexible enough to deform readily around each droplet as their intercalation between the primary surfactant molecules decreases both the polar head group interactions [18]. In this study, Tween 20 or Tween 80 was selected as potential surfactants and Transcutol/PG (1:1) as the co-surfactant system (Smix).

Previously, by the construction of ternary phase diagrams, it was demonstrated that when Tween 80 was used a surfactant with the same oil phase (Capmul MCM) and co-surfactant system (Smix), the optimal surfactant/co-surfactant (Smix) ratio was 4:2 [18]. Herein, we sought to confirm if the same ratio also applied when Tween 20 was used as the surfactant. Ternary phase diagrams were constructed by varying the Tween 20/Smix ratios to be: 1:2, 2:2, 3:2, and 4:2 (see Supplementary Materials Figure S2), for the selection of the ingredient amounts as required in order to avoid metastable formulations. From the ternary diagrams, it was confirmed that the 4:2 surfactant/Smix ratio was also optimal for Tween 20. Therefore, the optimal NE formulations that were selected for further experiments consisted by Capmul:surfactant + Smix:water at 8/44/48 and 10/44/46 ratios, where Smix consists of (Tween 80 or Tween 20):Transcutol:propylene glycol at a 4/1/1 ratio.

**Figure 3:**
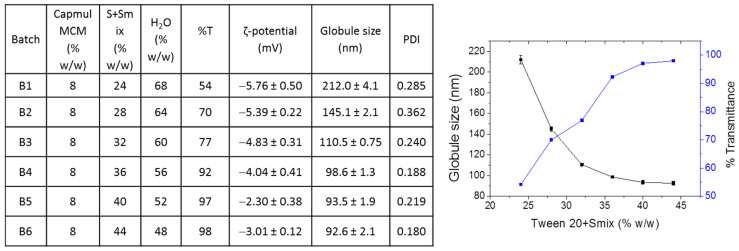
Physicochemical properties of the NEs composed of Tween 20 (as surfactant), with 8% Capmul MCM as the oil and various S + Smix percentages (from 24% up to 44%).

After confirming that the 4:2 ratio (for surfactant/co-surfactants) was also optimal when Tween 20 was used as the surfactant, several NEs using 8% Capmul MCM as the oil, and increasing concentrations of the surfactant (S) + co-surfactants (Smix) between 24 and up to 44%, were prepared in order to select the composition that acquired the optimal NE properties. As seen in Figure 3, composition B6 had the best properties with regard to the globule size distribution and transmittance (%).

**Figure 4:**
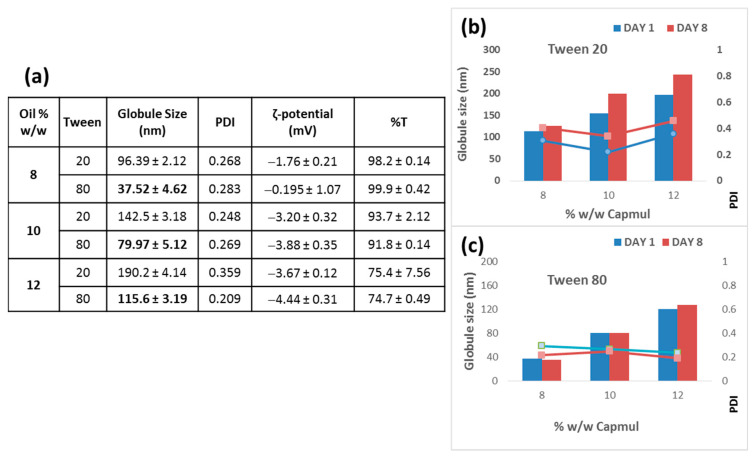
(**a**) Physicochemical properties of NEs composed of Tween 20 or Tween 80 (as the surfactant). (**b**) Stability of Tween 20 containing NEs (for 8 days at 25 °C). (**c**) Stability of Tween 80-containing NEs (for 8 days at 25 °C).

Thereby, we decided to use 44% concentration of S + Smix. Comparison of various NE formulations with Tween 80 or Tween 20 as the surfactant was then realized (after preparing various formulations) for the identification of the best surfactant. The results of their physicochemical properties and short-term physical stability study are presented in Figure 4.

As seen in Figure 4a, the NEs with Tween 80 had a significantly lower globule size for all oil concentrations tested, although their transmittance (%) was not affected by the different surfactants. Furthermore, Tween 80-containing NEs demonstrated higher stability (Figure 4c) during a preliminary 8 day at 25 °C stability study compared to the corresponding Tween 20-containing-NEs (Figure 4b), where a significant increase (*p* < 0.001) in globule size was observed after 8 day of storage at room temperature. In both types of NEs (with Tween 80 and Tween 20), when the oil concentration was increased so did the NE globule size. The last experiment showed that Tween 80-containing NEs had better properties and stability, and thereby Tween 80 was used as the surfactant in the following studies. For the latter decision, it was also taken into consideration that Tween 80 belongs to the class of non-ionic surfactants and is widely used since it is less toxic compared to ionic surfactants, and additionally, it is less affected by pH and ionic strength [19].

#### 3.2.3. Properties of BNN27-Loaded NEs

After the selection of the ingredients and the optimal S + Smix percent, BNN27-loaded NEs (BNEs) were formulated using the method described above. Two types of BNN27-loaded NEs were prepared: one with an oil phase of 8% (*w*/*w*) and the other of 10% (*w/w*), which were evaluated for their properties and stability.

**Figure 5:**
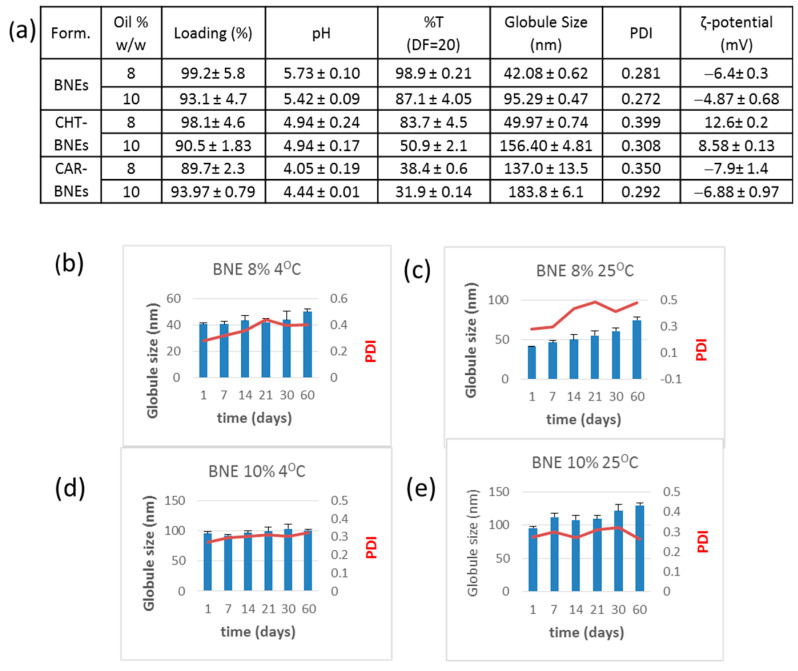
(**a**) Physicochemical properties of the BNN27-loaded NEs (BNEs) composed of Tween 80 (as the surfactant) and oil content of 8% or 10%. Some BNEs were coated with CHT (CHT-BNEs) or Carbopol (CAR-BNEs). (**b**,**c**) Stability of BNE with the 8% oil-phase during 60 days of storage at 4 °C and 25 °C, respectively. (**d**,**e**) Stability of BNE with the 10% oil-phase during 60 days of storage at 4 °C and 25 °C, respectively.

In general, a NE exhibits the characteristics of its external phase. There are several techniques for identifying the type of emulsion. Dilution studies are based on the fact that emulsions are soluble only in the liquid that forms their continuous phase. When diluted with water, no change was observed in the BNEs’ droplet size and clarity, indicating that the BNEs are oil-in-water emulsions. Additionally, neither phase separation nor creaming was observed after centrifugation of the NEs, suggesting the stability of the systems. The physicochemical properties and quantitative test data of the formulated BNEs are shown in Figure 5a. The pH of all BNEs is between 4.05 and 5.73, which is within the previously considered normal pH range of nasal fluid (3.5–6.4) [28]. However, it has recently been suggested that the pH of the nasal cavity is restricted in the range of 5.5–6.5 [29,30]. Thereby, the CAR-BNEs may most probably cause irritation following instillation on the nasal mucosa, while the CHT-coated ones may be considered as marginal. This is an issue that should be further explored in future studies.

The high degree of transparency of the non-coated BNEs verified that clear dispersions were formulated, while the CHT or CAR coated NEs had lower transmittance percentages due to the contribution of these components to turbidity. The low PDI of the non-coated BNEs indicates that they are a monodispersed system (Figure 5a).

All types of BNEs were also tested for drug content and found to demonstrate high BNN27 loading, ranging between approx. 90% and 99% of the amount of BNN27 used for their preparation (Figure 5a).

In the stability studies, the BNEs exhibited no precipitation of drug, creaming, phase separation, or flocculation on visual observation, and were found to be stable after centrifugation. When stored at 25 °C as well as at 4 °C (Figure 5b–e), only negligible changes in the quantitative parameters of the BNEs containing the 10%-oil phase were observed after 2 months of storage (Figure 5d,e). In contrast, the BNEs with the 8%-oil phase exhibited significant increases in their mean globule size (Figure 5b,c), especially during storage at 25 °C.

Similar stability studies were also carried out for the CHT- and CAR-coated BNEs (see Supplementary Materials Figure S3), where it was demonstrated that the BNEs with the 10%-oil phase were more stable for both types of coated BNEs compared with the corresponding BNE types with the 8%-oil phase. For this reason, BNN27-loaded NEs with 10% (*w*/*w*)-oil content (Capmul MCM) were used for the next in vitro and in vivo studies. The higher amount of oil phase in the BNEs would also provide the capability to load higher amounts of BNN27. Furthermore, it was previously reported the Capmul improved the NE-loaded drug permeation through the BBB barrier [21].

Additionally, the drug content of all BNE types studied for their stability was measured at all time points, and no significant decrease was demonstrated in the BNN27 content (or else no drug leakage occurred) in any of the tested BNE types (see Supplementary Materials Figure S4).

### 3.3. Cytotoxicity Evaluation

**Figure 6:**
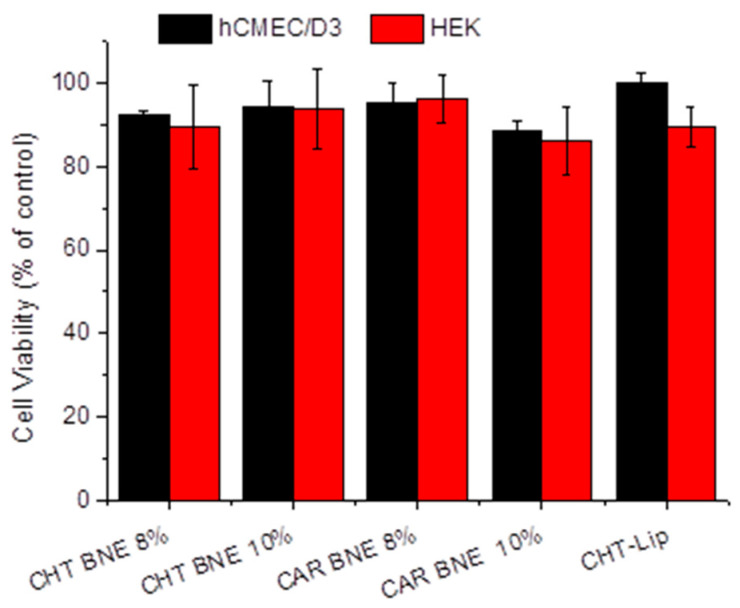
Cell viability of the hCMEC/D3 and HEK-294 cells following the 48 h incubation of various BNN27 nanoformulation types at a BNN27 concentration of 1 µM.

The cytotoxicity of the prepared NEs toward the hCMEC/D3 and HEK-294 cells was evaluated by the MTT method. As seen in Figure 6, no toxicity was observed in any of the cell lines studied after 48 h of incubation with BNEs.

### 3.4. TEM Morphology

**Figure 7:**
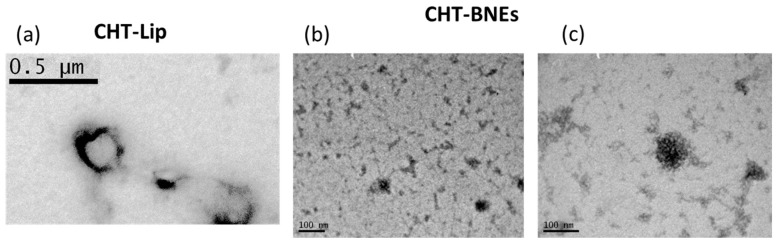
TEM micrographs of the CHT-coated (**a**) BNN27-loaded LIPs (PC/PG LIPs coated with 0.1% MMW CHT), (**b**) BNEs with 8%-oil phase, and (**c**) BNE with 10%-oil phase.

Transmission electron microscopy was performed to complete the characterization of the mucoadhesive nanoformulations. in Figure 7a, a polymeric chitosan membrane formed around the liposomal vesicles can be observed. It is interesting to highlight the different LIP mean-diameters observed by TEM and measured by DLS (which measures the hydrodynamic diameter), the last being more than two times smaller. For the BNEs, the TEM micrographs confirmed the differences in size of CHT-BMNEs with different percentages of the oil phase, as seen in Figure 7b,c. The latter differences are in agreement with the DLS measurements of the corresponding NE-types (Figure 5a).

### 3.5. Mucoadhesive Properties

**Figure 8:**
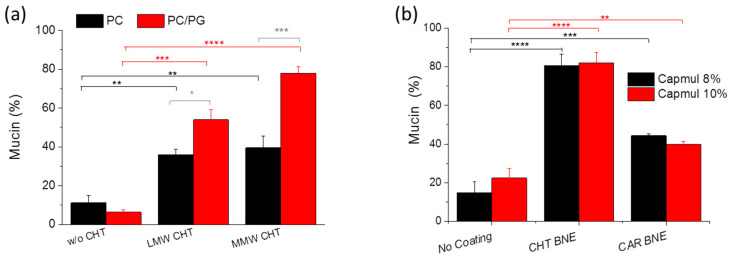
Mucoadhesive properties (expressed as the capability to adsorb mucin (% adsorbed)) of: (**a**) CHT-coated LIPs and (**b**) CHT- or CAR-coated NEs. Non-coated formulations were evaluated under identical conditions (in all cases) for comparison. *: *p* ≤ 0.05; **: *p* ≤ 0.01; ***: *p* ≤ 0.001; ****: *p* ≤ 0.0001.

Mucoadhesive properties were calculated as the percentage of mucin attached to LIPs or NEs. As seen in Figure 8, uncoated vesicles and NEs showed a small nonspecific mucoadhesion. As anticipated, PC/PG CHT-LIP is characterized by a strong positive surface charge (Table 1), which is why they exhibited significantly higher mucoadhesive properties compared to the PC CHT-LIPs. The uncoated PC/PG-LIPs were negatively charged and exhibited low mucoadhesive properties.

The adhesion process is rather complex and several theories have been proposed to explain the adhesion of polymeric materials [31,32,33,34]. Diffusion is one of the main theories proposed to describe mucoadhesion, which includes the action of polymer-chain entanglement [35]. The diffusion theory states that inter-penetration of the polymer and mucin chains may lead to prolonged mucosal and mechanical adhesion. According to the diffusion theory, we expect that since the MW of MMW-CHT is nearly 10 times greater than that of LMW-CHT, the contribution of the natural entanglement to the adhesion between MMW-CHT and mucin should be much stronger than that between LMW-CHT and mucin. Indeed, as seen in Figure 8a, MMW-CHT conferred significantly higher mucoadhesive properties to the PC/PG coated LIPs compared to LMW-CHT. High molecular weight chitosan (HMW) was not used in our studies because it has been previously shown to confer lower mucoadhesive properties than MMW, a result attributed to the higher probability of the very long chains of HMW-CHT to bend, leading to fewer available amino groups, and thus providing a lower positive charge for interaction [36].

Concerning the mucoadhesive properties of the coated BNEs, as seen in Figure 8b, both polymers, CHT and CAR, resulted in a significant increase in the mucoadhesive properties of non-coated BNEs, however, the mucoadhesive properties of the CHT-coated BNEs were about two times higher than those of the corresponding CAR-coated BNEs. Indeed, it has also been previously observed that the mucosal capacity of CHT is significantly higher than the relevant properties of CAR [37,38]. Finally, the oil content (percent) of the BNEs did not seem to have any effect on the mucoadhesive properties of the BNEs, at least for BNEs with 8% or 10% (*w*/*w*) oil.

### 3.6. In Vitro and In Vivo Studies

The permeability of the formulation-incorporated RHO across a cellular model of the BBB as well as the brain disposition of BNN27 following the intranasal administration of BNN-loaded formulation to mice were evaluated in the last part of the current study, in order to compare the various types of formulations. It should be clarified at this point that the hCMEC/D3 monolayer is considered as a model of “intact” BBB (with tight connections between the cells), while the nasal CNS barrier is proposed to be more ‘‘leaky’’ because of continuous neuron turnover [29,39]. Nevertheless, we thought that it would be interesting to conduct comparative studies of the two formulation types under identical conditions in the hCMEC/D3 model and also after intranasal administration (in vivo).

#### 3.6.1. In Vitro Permeability across hCMEC/D3 Monolayer

**Figure 9:**
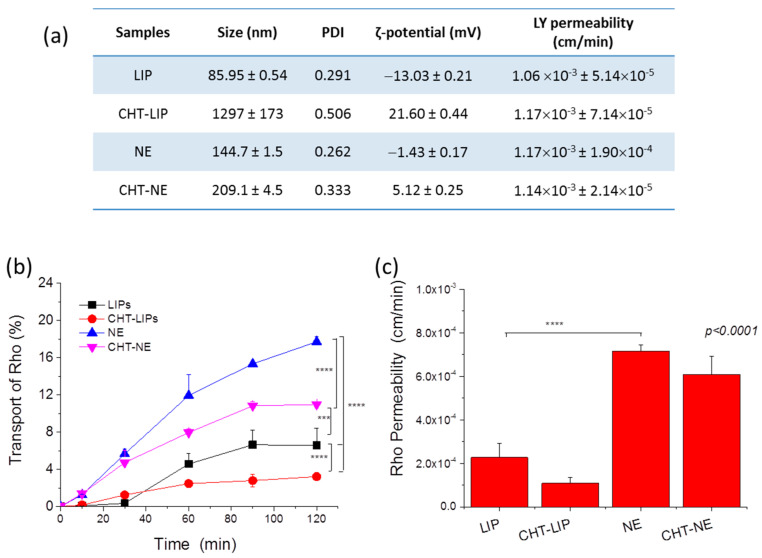
(**a**) Physicochemical properties of formulations used in the BBB-model permeability study. (**b**) Transport of formulation-associated RHO (%) across the monolayer with time (following incubation with LIP and NE formulations at 0.2 µM RHO concentration). (**c**) Permeability values of formulations (formulation-associated RHO permeability) across the hCMEC/D3 monolayer (calculated from the results of Figure 9b). ***: *p* ≤ 0.001; ****: *p* ≤ 0.0001.

For the permeability study, formulations incorporating 1 mol% RHO as a lipophilic drug model were used. LIP (PC/PG) and CHT-coated LIP (coated with MMW CHT) as well as NE and CHT-NE with 10% Capmul were prepared; the physicochemical properties of the formulations are presented in Figure 9a.

During monolayer formation, the TEER of the monolayers was measured and was found to gradually increase from 31.5 Ω × cm^2^ (at day 3) to 111.1 Ω × cm^2^ (after simvastatin treatment), in agreement with previous results [23,24]. Lucifer yellow (LY, a marker for paracellular transport) was added in all of the monolayer experiments (wells) and its permeability was measured as a method to identify any toxicity of the samples toward the monolayer integrity, which would thus impair the accuracy of the transport results. As seen in Figure 9a, LY permeability was practically un-influenced by the samples, ranging between 1.06 × 10 ^3^ and 1.24 × 10 ^3^ cm/min for all samples, being in good agreement with the previously reported values [23,24].

In preliminary cytotoxicity studies, it was confirmed that all the formulations used were non-cytotoxic toward the hCMEC/D3 cells following 2 h incubation with the cells at a final RHO-lipid concentration of 0.2 µM (see Supplementary Materials Figure S5), as also seen for the BNN27-incorporating formulations at a 1 µM concentration after 48 h (Figure 6). However, the substantial difference in the cytotoxicity of the two formulation types is a matter that needs to be highlighted. Indeed, RHO-incorporating LIPs (non-coated and coated) did not confer any cytotoxicity toward the hCMEC/D3 cells at 5 µM concentration (of RHO) after 48 h (Figure S5a), whereas the RHO-incorporated NEs diminished the cells even at 10 times a lower concentration (of RHO) and only 2 h incubation (Figure S5b).

Concerning the comparison between LIPs and NEs, interestingly, the translocation of NE-associated RHO and the corresponding permeability values were approx. three times higher for the corresponding values of LIPs. This is a very interesting finding, especially since such differences between LIPs and NEs have not been previously reported (to the best of our knowledge).

Concerning the effect of coating, the transport of the coated-nanoformulations (Figure 9b) as well as the corresponding permeability of the formulation-associated RHO (Figure 9c) were found to be lower than the corresponding values of the control (non-coated) nanoformulations, for both formulation types (LIPs and NEs) (Figure 9b), although in some cases, the differences noted were not statistically significant. This fact can be attributed to the adhesion of the CHT LIPs or NE droplets to the monolayer, resulting in delayed permeability. Free RHO (micellar solution) did not show any difference compared to LIP-RHO (not shown), while compared to the NEs, its permeability was significantly lower, which is in good agreement with the previously reported results [40].

#### 3.6.2. BNN27 In Vivo Intranasal Delivery Studies

**Figure 10:**
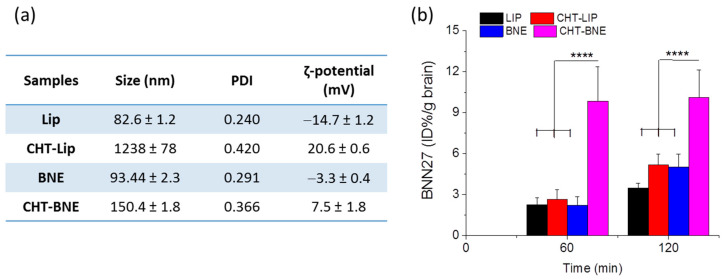
(**a**) Physicochemical properties of the formulations used in the in vivo disposition study. (**b**) Brain disposition of BNN27 1 and 2 h post-intranasal administration of the BNN27 loaded liposomal and NE formulations. Each value is the mean of at least three independent samples. ****: *p* ≤ 0.0001.

The calibration curve used for the calculation of the BNN27 concentration in the brain samples is presented in Figure S6 (see Supplementary Materials). As seen in Figure 10, the brain disposition of BNN27 after intranasal administration of BNN27 nanoformulations to C57BL/6J mice showed that CHT-BNEs achieved the highest levels of BNN27 in the brain compared to all of the other formulations used. Specifically, the BNN27 levels in the brain 1 h after nasal administration of CHT-BNE were more than 3.5 times higher than the corresponding concentrations after the administration of LIPs (coated or non-coated) or non-coated BNEs. At 2 h post-administration, the differences in brain disposition were lower, since all other formulations demonstrated increased brain concentrations of BNN27 at this time point (compared to the 1 h time point), except for CHT-BNE, but the BNN27 concentration after administration of CHT-BNEs was still more than 2-fold higher compared to all other nanoformulations (Figure 10b).

As demonstrated, 2 h post-administration, the non-coated BNEs achieved a higher BNN27 brain concentration compared to non-coated LIPs (although the difference was not statistically significant); however, nasal administration of CHT-coated nanoformulations resulted in higher brain concentrations of BNN27 in most cases, with the exception of LIPs at 1 h post-administration. Another very interesting observation from the data of Figure 10b is the very rapid brain disposition of CHT-BNE-associated-BNN27, which reached the highest BNN27 brain concentration already 1 h post-administration, in contrast to all of the other nanoformulations that demonstrated the highest BNN27 brain concentrations 2 h post-administration.

It has been reported that the natural biodegradable polymer chitosan may enhance the penetration and absorption of drugs through the nasal mucosa and may also delay mucociliary clearance [41], thus increasing the absorption of drugs after intranasal delivery. Additional advantages of coating with CHT are its excellent biocompatibility as well as the fact that it is a well-tolerated polymer [42]. Several studies have confirmed a double role for CHT as a coating of NEs, proving that chitosan-coated NEs conferred the highest fluxes and nasal mucosa permeability compared to the corresponding uncoated NEs [10].

## 4. Conclusions

Herein, two types of nanoformulations, LIPs and NEs, were developed using biocompatible ingredients, optimized for drug loading, stability, and mucoadhesive properties, and evaluated as nanoformulations for the nose-to-brain delivery of BNN27. The effect of the CHT-coating was evaluated for both formulation types.

The results showed that NEs (consisted of Capmul MCM, Tween 80, Transcutol, and propylene glycol) had significantly higher in vitro BBB-crossing capability compared to LIPs (consisted of PC and PG) (Figure 9); however, the CHT-coating resulted in a slight decrease in the BBB monolayer transport for both formulation types, perhaps due to the adhesion of CHT-coated vesicles/globules on the apical side of the monolayer, thus preventing their transport to the basal side. In agreement with the monolayer permeability results, BNEs demonstrated significantly higher brain disposition of BNN27 compared to the BNN27-loaded LIPs (Figure 10). This result may be attributed to the previously reported effect of Capmul MCM to enhance drug permeability across the nasal mucosa and thus the brain disposition of drugs [21,43]. Furthermore, in vivo, the CHT-coating conferred enhanced brain concentrations of BNN27 for both formulation types (NEs and LIPs). In the case of LIPs, the effect of the CHT-coating became evident only at 2 h post-administration, suggesting that prolonged retention of the coated LIPs at the site of administration (nasal mucosa) is probably implicated in the higher BNN27 brain amounts, as also proposed in other cases [44]. In fact, it has previously been reported that CHT increased NE permeability, which was attributed to its penetration enhancing properties by the transient opening of tight junctions [45]. Higher permeability of a CHT NE formulation was also suggested to explain the higher AUC and shorter Tmax of the NE-associated drug (in the brain) compared to i.v. or nasal administration of the drug solution [46]. Interestingly, CHT-BNE demonstrated very high brain disposition of BNN27 already at 1 h post-administration, which was not the case for the non-coated BNE. The latter result suggests that perhaps the presence of CHT on the much smaller globules of the BNE (compared to LIPs) has a more direct effect on the permeability and brain disposition of the drug, and not only on the retention of the drug on the nasal mucosa. The particle size of the formulations may also be important. Indeed, it has been suggested that particle size and not the CHT coating is the most important determining factor for NE brain disposition following nasal delivery, and that formulations with sizes around 100 nm demonstrate prolonged residence in the nasal cavity, whereas NEs of larger sizes have faster clearance. It was additionally proven by imaging methods that NEs with globule sizes of 100 nm were transported through the trigeminal and the olfactory nerves to the olfactory bulb, while larger NE transport through the nose-to-brain route was lower due to higher mucociliary clearance [47]. This last report could perhaps explain the significant difference between the CHT LIPs and CHT BNE regarding the BNN27 brain disposition. However, as highlighted above, it has to be emphasized that the hydrodynamic mean diameters of liposomes (especially the ones coated with CHT) measured by DLS were much larger than their actual sizes, as proven from the TEM micrographs (Figure 7a). Nevertheless, the CHT LIPs had significantly larger diameters compared to the CHT BNEs (Figure 7), suggesting that this may result in higher mucociliary clearance and reduced transport through the nose-to-brain route, according to previous findings [47].

A direct comparison of LIPs and NEs for nose-to-brain delivery of drugs has never been evaluated before; therefore, we cannot discuss the current results with respect to previous studies. We identified two studies reporting a direct comparison of LIPs and NEs for other drug delivery applications: one that compared the skin delivery of retinyl palmitate [48], and another in which the two formulation types were compared for the brain delivery of melatonin after i.v. injection [49]. In the first report, the cumulative amount of drug that permeated through human skin after 38 h was 6.67 ± 1.58 mg, and 4.36 ± 0.21 mg for NEs and LIPs, respectively, while the NE flux was significantly higher than that of the LIPs. In the same study, it was reported that the LIPs resulted in significantly higher skin retention compared to NEs, while the NEs disrupted the skin more [48]. In the second study, although the melatonin bioavailability was similar for LIPs and NEs, a NE formulation containing medium chain triglycerides as the oil phase resulted in a higher fraction of animals that reached the critical concentration of the drug in brain extracellular fluid (and also faster) compared to the LIPs [49].

In summary, the CHT-BNE formulation developed herein was demonstrated to confer faster and higher nose-to-brain delivery of BNN27 compared to CHT-LIPs. Such NEs could be considered as alternative systems for the brain delivery of lipophilic drugs following intranasal administration. Nevertheless, extended biocompatibility and toxicity studies are required to exclude any potential toxicity issues in relation to the cytotoxicity differences between the LIPs and NEs above-mentioned due to the high surfactant content of NEs.

## Figures and Tables

**Table 1:** Physicochemical properties of the CHT-coated LIPs. Effect of lipid membrane composition and CHT type and amount.

Lipos	CHT MW-CHT/LIP (*w*/*w*)	Mean Diameter (nm)	PDI	ζ-Potential (mV)	Coating Efficiency (%)
PC	Non-coated	78.1 ± 1.15	0.281	−3.48 ± 0.49	-
	Low-0.1	123.7 ± 2.17	0.398	3.65 ± 0.27	3.5 ± 0.2
	Med.-0.1	158.8 ± 6.27	0.405	4.57 ± 0.18	4.8 ± 0.6
PC/PG	Non-coated	83.01 ± 1.36	0.263	−12.4 ± 0.689	-
	Low-0.1	822 ± 21.54	0.452	15.2 ± 0.47	79.9 ± 1.5
	Med.-0.1	1147 ± 86.27	0.447	23.1 ± 1.41	85.1 ± 4.5
	Med.-0.3	2563.5 ± 109.6	0.592	24.4 ± 1.273	78.2 ± 0.7
	Med.-0.5	5155 ± 51.24	0.617	29.4 ± 0.689	81.4 ± 1.8

## Supplementary Materials

The following supporting information can be downloaded at: https://www.mdpi.com/article/10.3390/pharmaceutics15020419/s1, Figure S1: Representative calibration curves for quantification of BNN27 in (a) Solutions (b) LIPs and (c) in Oil ingredients and Nanoemulsions; Figure S2: Ternary Diagrams of NEs composed of Capmul MCM as oil, Tween 20 and Smix as surfactant/co-surfactants system, at ration 1:2, 2:2, 3:2 and 4:2, and water. b. Globule size of the NEs having different S/Smix ratios; Figure S3: (a) and (b), Stability of CHT-coated BNE with 8% oil-phase during 60 day storage at 4 °C and 25 °C, respectively. (c) and (d). Stability of CHT-coated BNE with 10% oil-phase during 60 day storage at 4 °C and 25 °C, respectively. (a1) and (b1) Stability of Carbopol (CAR)-coated BNEs with 8% oil-phase containing NEs during 60 day storage at 4 °C and 25 °C, respectively. (c1) and (d1) Stability of CAR-coated BNEs with 10 % oil-phase during 60 day storage at 4 °C and 25 °C, respectively; Figure S4: Stability of drug content (%) of BNEs, CHT-coated BNEs and CAR-coated BNEs, during 60 day storage at 4 °C (a and c) or 25 °C (b) and (d), for BNEs with 8% oil-phase (a) and (b) or 10% oil-phase (c) and (d); Figure S5: Cell viability of hCMEC/D3 cells following 48 h (a) or 2 h (b) incubation of RHO-incorporating nanoformulations, at various RHO (and associated formulation) concentrations, as seen in the graph inserts.

## Abbreviations

Ach: achetylocholine; BBB: blood–brain barrier; CHT: chitosan; CNS: central nervous system; D/L: drug to lipid ratio; DHEA: dehydroepiandrosterone; DLS: dynamic light scattering; FBS: fetal bovine serum; GABA: γ-amino butyric acid; HEK: human embryonic kidney cells; hCMEC/D3: immortalized human cerebral microvascular endothelial cells; HMW: high molecular weight; LDE: laser Doppler electrophoresis; LIP: liposomes; LMW: low molecular weight; MMW: medium molecular weight; LY: Lucifer yellow; MTT: 3-(4,5- dimethylthiazol-2-yl)-2,5 diphenyltetrazolium bromide; MW: molecular weight; NE: nanoemulsion; NGF: nerve growth factor; PBS: phosphate buffered saline; PC: 1,2-distearoyl-sn-glycerol-3-phosphatidylcholine; PG: 1,2-distearoyl-sn-glycero-3-phospho-(1′-rac-glycerol) (sodium salt); p75NTR: p75 neurotrophin receptor; RHO: Lissamine Rhodamine B phosphatidylethanolamine; TEER: transendothelial electrical resistance; TEM: transmission electron microscopy; TrkA: tropomyosin receptor kinase A.
