# Supplementary material for: Development and Comparative In Vitro and In Vivo Study of BNN27 Mucoadhesive Liposomes and Nanoemulsions for Nose-to-Brain Delivery
Source: Pharmaceutics. 2023 Jan 27;15(2):419. doi: 10.3390/pharmaceutics15020419 (PMC9967044; doi:10.3390/pharmaceutics15020419)
Supplement: Supplementary file 1 [file pharmaceutics-15-00419-s001.zip › pharmaceutics-2175396-supplementary-done.pdf]

pharmaceutics

MDPI

Supplementary Materials:

Development and Comparative In Vitro and In Vivo Study of BNN27 Mucoadhesive Liposomes and Nanoemulsions for Nose-to-Brain Delivery

Maria Kannavou, Kanelina Karali, Theodora Katsila, Eleni Siapi, Antonia Marazioti, Pavlos Klepetsanis, Theodora Calogeropoulou, Ioannis Charalampopoulos and Sophia G. Antimisiaris

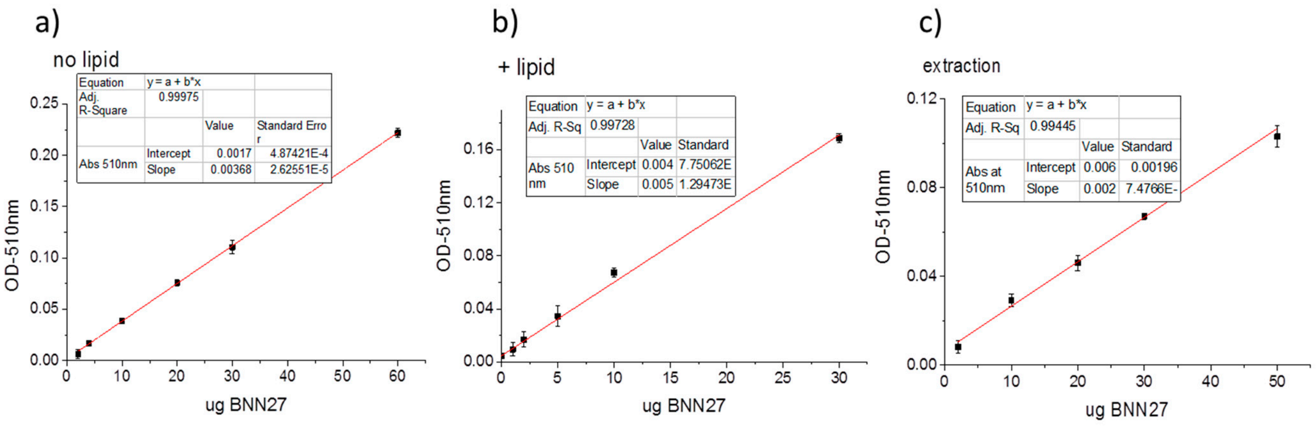

Figure S1. Representative calibration curves for quantification of BNN27 in (a) Solutions (b) LIPs and (c) in Oil ingredients and Nanoemulsions

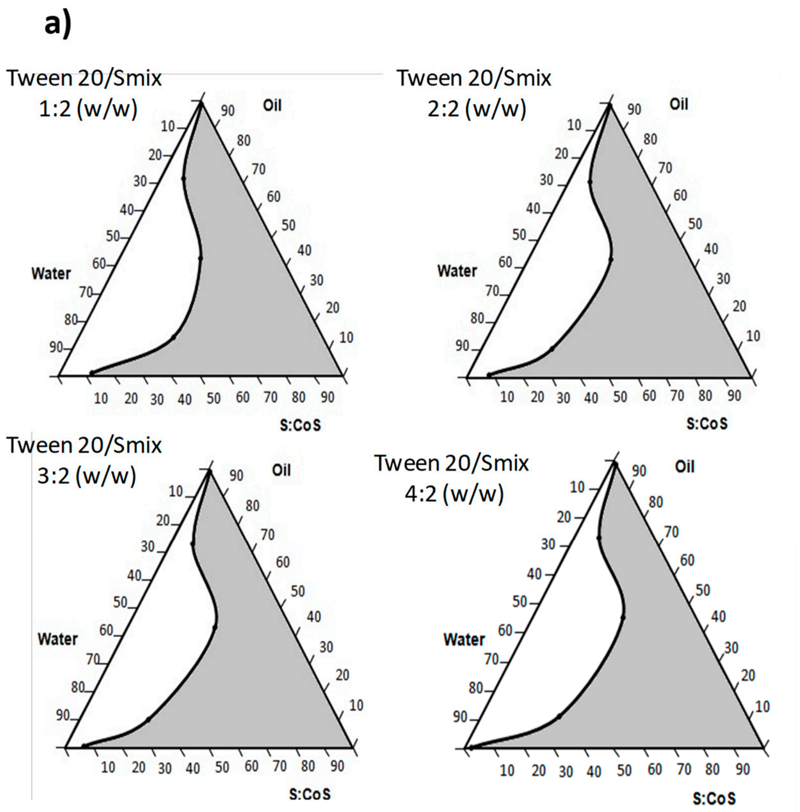

b)

NEs globule size at different Surfactant/Smix ratios (Oil:S+Smix 3:7 w/w)

Tween 20:Smix (w/w)	Globule Size (nm)
1:2	259.4 ± 1.626
2:2	201.5 ± 4.614
3:2	110.9 ± 2.141
4:2	71.04 ± 0.339

Figure S2. Ternary Diagrams of NEs composed of Capmul MCM as oil, Tween 20 and Smix as surfactant/co-surfactants system, at ration 1:2, 2:2, 3:2 and 4:2, and water. b. Globule size of the NEs having different S/Smix ratios

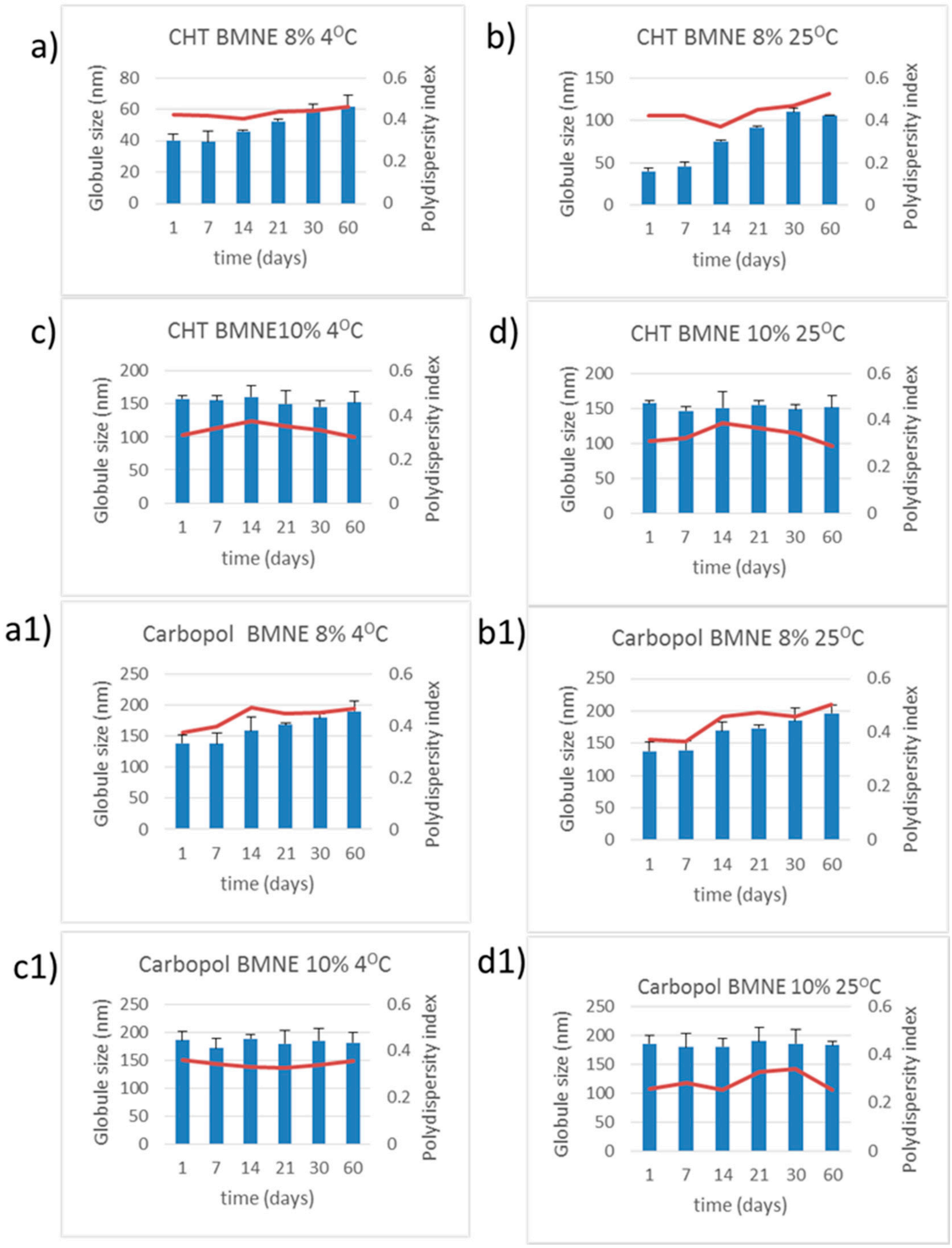

Figure S3. (a) and (b), Stability of CHT-coated BNE with 8% oil-phase during 60 day storage at 4°C and 25°C, respectively. (c) and (d). Stability of CHT-coated BNE with 10% oil-phase during 60 day storage at 4°C and 25°C, respectively. a1) and b1) Stability of Carbopol (CAR)-coated BNEs with 8% oil-phase containing NEs during 60 day storage at 4°C and 25°C, respectively.c1) and d1) Stability of CAR-coated BNEs with 10 % oil-phase during 60 day storage at 4°C and 25°C, respectively

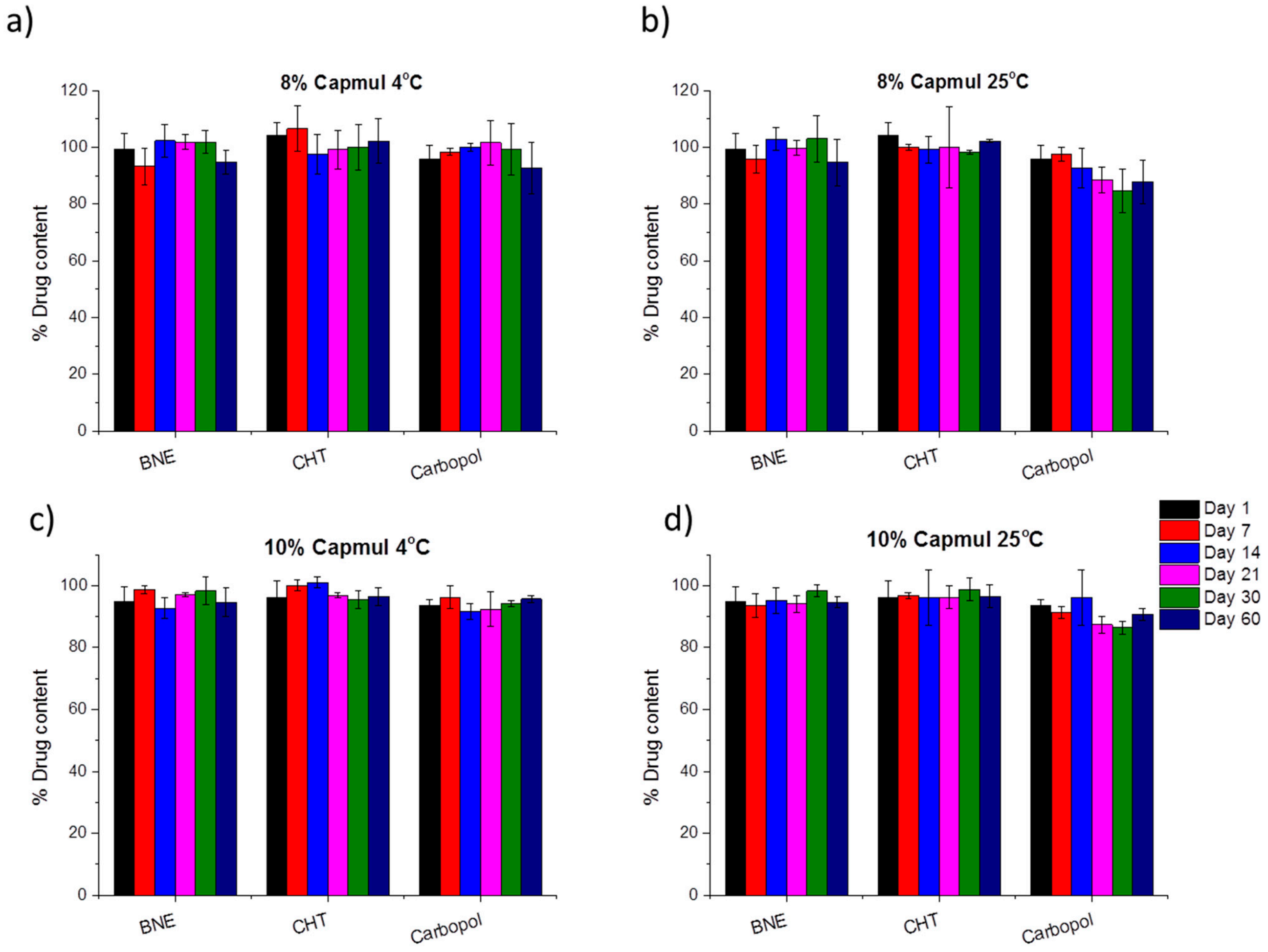

Figure S4. Stability of drug content (%) of BNEs, CHT-coated BNEs and CAR-coated BNEs, during 60 day storage at 4°C (a and c) or 25°C (b) and (d), for BNEs with 8% oil-phase (a) and (b) or 10% oil-phase (c)and (d).

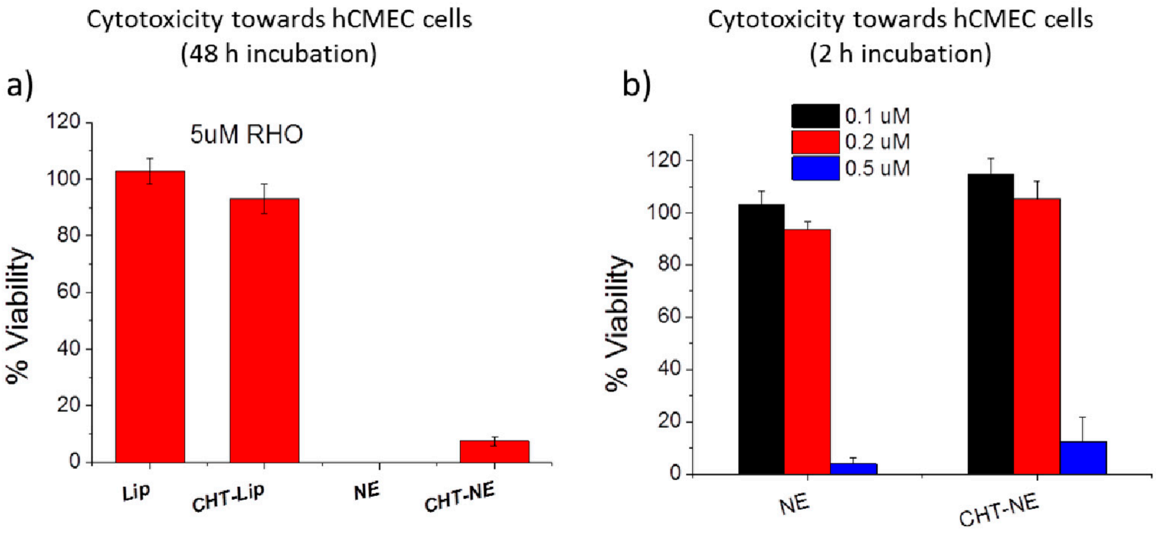

Figure S5.Cell viability of hCMEC/D3 cells following 48 h(a) or 2 h (b) incubation of RHO-incorporating nanoformulations , at various RHO (and associated formulation) concentrations, as seen in the graph inserts.

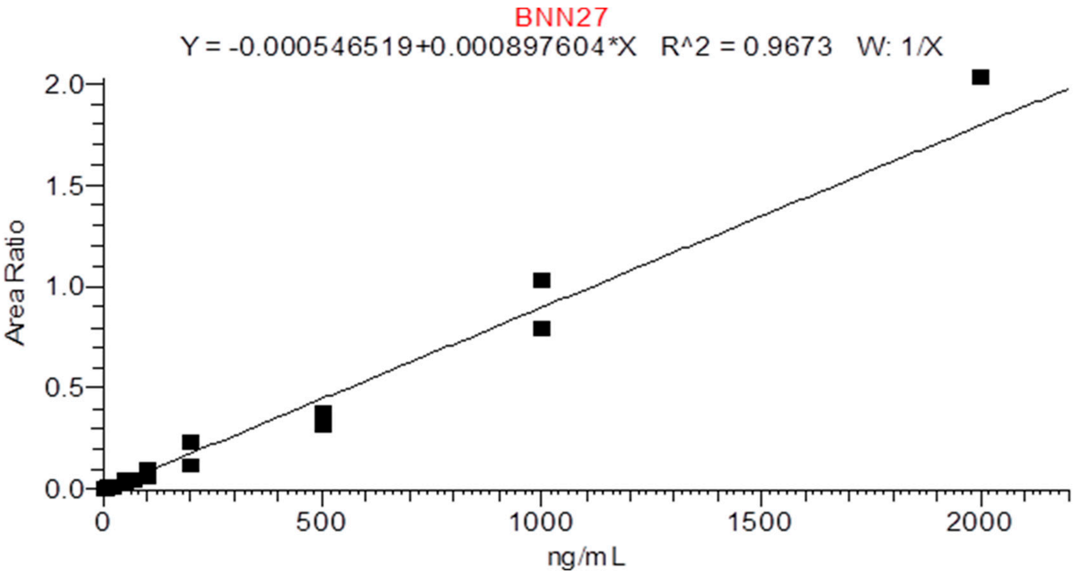

Figure S6. Calibration curve for quantification of BNN27 in processed brain tissues by LC-MSn method, Details of method and sample processing are mentioned in Materials and Methods.
